# Alterations in resting-state network dynamics along the Alzheimer’s disease continuum

**DOI:** 10.1038/s41598-020-76201-3

**Published:** 2020-12-15

**Authors:** D. Puttaert, N. Coquelet, V. Wens, P. Peigneux, P. Fery, A. Rovai, N. Trotta, N. Sadeghi, T. Coolen, J.-C. Bier, S. Goldman, X. De Tiège

**Affiliations:** 1 Laboratoire de Cartographie fonctionnelle du Cerveau (LCFC), UNI-ULB Neuroscience Institute, Université libre de Bruxelles (ULB), Brussels, Belgium; 2 Department of Functional Neuroimaging, Service of Nuclear Medicine, CUB Hôpital Erasme, Université libre de Bruxelles (ULB), Brussels, Belgium; 3 Neuropsychology and Functional Neuroimaging Research Unit (UR2NF), Center for Research in Cognition and Neurosciences (CRCN), UNI-ULB Neuroscience Institute, Université libre de Bruxelles (ULB), Brussels, Belgium; 4 Service of Neuropsychology and Speech Therapy, CUB Hôpital Erasme, Université libre de Bruxelles (ULB), Brussels, Belgium; 5 Department of Radiology, CUB Hôpital Erasme, Université libre de Bruxelles (ULB), Brussels, Belgium; 6 Department of Neurology, CUB Hôpital Erasme, Université libre de Bruxelles (ULB), Brussels, Belgium

**Keywords:** Molecular biology, Neuroscience, Physiology, Psychology, Biomarkers, Diseases, Medical research, Molecular medicine, Neurology

## Abstract

Human brain activity is intrinsically organized into resting-state networks (RSNs) that transiently activate or deactivate at the sub-second timescale. Few neuroimaging studies have addressed how Alzheimer's disease (AD) affects these fast temporal brain dynamics, and how they relate to the cognitive, structural and metabolic abnormalities characterizing AD. We aimed at closing this gap by investigating both brain structure and function using magnetoencephalography (MEG) and hybrid positron emission tomography-magnetic resonance (PET/MR) in 10 healthy elders, 10 patients with subjective cognitive decline (SCD), 10 patients with amnestic mild cognitive impairment (aMCI) and 10 patients with typical Alzheimer’s disease with dementia (AD). The fast activation/deactivation state dynamics of RSNs were assessed using hidden Markov modeling (HMM) of power envelope fluctuations at rest measured with MEG. Correlations were sought between temporal properties of HMM states and participants' cognitive test scores, whole hippocampal grey matter volume and regional brain glucose metabolism. The posterior default-mode network (DMN) was less often activated and for shorter durations in AD patients than matched healthy elders. No significant difference was found in patients with SCD or aMCI. The time spent by participants in the activated posterior DMN state did not correlate significantly with cognitive scores, nor with the whole hippocampal volume. However, it correlated positively with the regional glucose consumption in the right dorsolateral prefrontal cortex (DLPFC). AD patients present alterations of posterior DMN power activation dynamics at rest that identify an additional electrophysiological correlate of AD-related synaptic and neural dysfunction. The right DLPFC may play a causal role in the activation of the posterior DMN, possibly linked to the occurrence of mind wandering episodes. As such, these data might suggest a neural correlate of the decrease in mind wandering episodes reported in pathological aging.

## Introduction

Alzheimer’s disease (AD) is a degenerative brain disease that is the most common cause of dementia, accounting for 60–70% of all cases. Worldwide, about 50 million people are affected by dementia and its prevalence is expected to reach 82 million in 2030 (https://www.who.int/en/news-room/fact-sheets/detail/dementia).

Pathological changes begin years before symptoms onset in AD and are characterized by an abnormal accumulation of extracellular aggregates of amyloid-β peptides leading to senile plaques and intracellular aggregates of hyperphosphorylated tau proteins leading to neurofibrillary tangles (see Refs.^1, 2^ for a description of the progressive cerebral deposition). Although debated (see, e.g., Refs.^3–5^), it is hypothesized that these protein aggregates generate abnormal neural/synaptic activity leading to neural death^6^, and act synergistically to drive, by cell-to-cell transmission, the pathological progression of AD from the hippocampus to connected brain areas mainly encompassing the default-mode network (DMN). The DMN is composed of a set of brain regions (i.e., precuneus, posterior cingulate cortex (PCC), medial prefrontal cortex (MPFC), temporoparietal junctions (TPJs) and hippocampus) whose coordinated activity is higher when the mind is not engaged in specific behavioral tasks (i.e., during spontaneous cognitive processes at undirected rest) and lower during focused attention on the external environment or during goal-directed tasks^7^. Interestingly, there is a significant overlap between DMN regions and brain areas with high levels of amyloid-β and tau protein deposition, brain atrophy and hypometabolism in AD, which suggests that the DMN is particularly vulnerable to AD pathology^8^.

The evolution towards an established dementia due to AD is progressive, so preclinical (e.g., subjective cognitive decline (SCD)) and predementia (e.g., mild cognitive impairment (MCI)) stages should be identified before clinical criteria of dementia are fully developed. SCD is defined as a self-perceived decline in cognitive capacity despite normal performance at neuropsychological assessment and no significant impact on everyday activities^9^. MCI is characterized by concerns regarding a change in cognitive capabilities confirmed by an objective impairment in one or more cognitive function(s) without an impact on everyday activities^10^ (for a recent review, see Ref.^11^). These two preclinical/predementia stages could represent a critical period during which disease-modifying treatments could slow down or even stop the degenerative progression towards AD and, consequently, dramatically improve the patients' quality of life.

Resting-state functional magnetic resonance imaging (rsfMRI), which measures the spontaneous fluctuations of blood oxygenation level-dependent (BOLD) signals in brain regions at rest (i.e., in the absence of any explicit or goal-directed task), has been considered as an emerging AD-continuum marker (for reviews, see Refs.^12, 13^). Spontaneous BOLD signals typically covary between different brain areas, which is thought to reflect large-scale resting-state functional networks (i.e., the so-called resting-state networks (RSNs)) whose anatomical architecture is close to task-based functional networks^14, 15^. Among changes in RSNs uncovered by rsfMRI, evidence for AD-related alterations of DMN resting-state functional connectivity (rsFC) appears as a promising in-vivo marker of AD and its preclinical/predementia stages^16–18^. Still, the use of fMRI along the AD continuum suffers from two important pitfalls. First, fMRI provides an indirect measure of neuronal activity via the neurovascular coupling. However, vascular changes (e.g., in vessel hemodynamics, angiogenesis; for a review, see Ref.^19^) are an early preclinical feature of AD pathology leading to modifications in cortical blood flow even before the onset of clinical symptoms^20^. Thus, it cannot be ruled out that parts of rsfMRI changes observed along the AD continuum actually reflect vascular rather than neuronal changes per se. Second, the temporal resolution of fMRI is relatively poor (at the level of the second), which strongly impedes the characterization of the temporal and spectral dynamics of human brain activity. This means that subtle AD-related changes in neural dynamics will less likely be detected with fMRI^21^. This explains why electrophysiological techniques such as magnetoencephalography (MEG) are increasingly recognized as potentially valuable markers in AD^22, 23^. MEG is a non-invasive neuroimaging technique measuring the extra-cranial magnetic fields produced by electrical neural activity^24^. Contrary to fMRI, it provides a direct measure of neural activity together with an exquisite temporal resolution (of the order of milliseconds), allowing proper investigation of the temporal oscillatory dynamics of human brain activity, and is not influenced by age- and pathology-related changes in vascular coupling. MEG studies demonstrated that RSNs (including the DMN) emerge from large-scale correlation patterns in the slow fluctuations of band-limited power envelope estimated over several minutes, particularly in the alpha and beta frequency bands, confirming the neural basis of RSNs uncovered by rsfMRI^25–31^. In addition, the high temporal resolution of MEG allows uncovering the transient dynamics of rsFC within and across RSNs. The DMN appears to play a central role in this dynamic functional integration of brain networks at rest^28, 32^. Importantly, these supra-second RSN dynamics could emerge from rapid transitions between recurrent brain states^32, 33^.

To the best of our knowledge, only one MEG study investigated so far the AD-related changes in the fast state dynamics underlying RSN activity^34^, using an approach based on hidden Markov modeling (HMM). HMM identifies transient state configurations by classifying distinct patterns of power envelope (co)variance consistently repeating in time (for a review, see Ref.^35^). From MEG data, about 6–8 transient recurring states lasting 50–200 ms are typically disclosed with a spatial topography quite similar to that of the main RSNs^33, 34, 36, 37^. Results obtained in AD patients demonstrated that a state of increased power in the DMN (including bilateral inferior parietal lobes, medial prefrontal cortex and lateral temporal cortices) was visited less often and for shorter periods of time in participants with AD than in matched healthy subjects^34^. This finding suggests that spontaneous DMN activation is destabilized by AD neurodegeneration^34^. In that study, though, the diagnosis of AD was clinical, and patients with SCD or MCI were not investigated^34^. Furthermore, the reported DMN activation state did not cover the typical posterior midline cortices (i.e., the precuneus and PCC)^34^, possibly due to methodological issues related to source reconstruction^38^.

In the present multimodal neuroimaging study, we applied HMM on MEG power envelope activity^33^ to further characterize the alterations in RSN state dynamics along the AD continuum, and to determine their link with changes in cognitive function, hippocampal volume and regional brain glucose metabolism. Resting-state neuromagnetic activity was investigated in healthy elders and in patients with SCD, aMCI and typical AD. MEG power envelopes were reconstructed using Minimum Norm Estimation (MNE) as it allows uncovering the characteristic posterior midline cortices of the DMN^38^. We expected (1) to replicate the findings of an abnormal DMN activation dynamics in AD^34^, (2) that reconstruction of the posterior midline cortices of the DMN would bring novel information about AD-related changes in the dynamic stability of that core human brain network, (3) that inclusion of preclinical and predementia stages would highlight subtle modifications in the dynamics of human brain activity at rest that could serve as potential markers of the evolution toward an AD pathology, and (4) that cross-modal correlation analyses would provide novel insights into the understanding of the pathophysiological mechanisms at the origin of these AD-related changes.

## Results

Forty participants were included in this study: 10 healthy elders (age mean and standard deviation: 70.2 ± 5.55 years; 6 females), 10 patients with SCD (71.7 ± 7; 4 females), 10 patients with aMCI (74 ± 5.47; 6 females) and 10 patients with AD (72.3 ± 7.7; 6 females). All patients with AD were characterized by abnormal levels of amyloid-β and tau proteins in the CSF. The participant groups did not differ in age, gender ratio, years of education and handedness. Their demographic and clinical assessment data are detailed in Table 1.

**Table 1:** Participants' demographic and clinical data.

	HE (n = 10)	SCD (n = 10)	aMCI (n = 10)	AD (n = 10)	*p* value
**Age**, *years*	70.2 ± 5.55	71.7 ± 7	74 ± 5.47	72.3 ± 7.7	0.63
**Gender**, *females*	6	4	6	6	0.77
**Education**, *years*	14.4 ± 3.83	16.2 ± 5.26	12.5 ± 4.08	12.9 ± 3.17	0.2
**GDS**	1.5 ± 2.61	4.9 ± 3.28	3.8 ± 3.73	3.66 ± 4.18	0.25
**CDR**	0 ± 0	0 ± 0	0.5 ± 0	0.78 ± 0.26	**< 0.001**
**CSF biomarkers**					
CSF Aβ, *pg/mL*	na	na	na	253.8 ± 112.1	**–**
CSF tau, *pg/mL*	na	na	na	707 ± 334.1	**–**
CSF p-tau, *pg/mL*	na	na	na	92.1 ± 31.2	**–**
**sMRI biomarker**					
WHV, *mm*^3^	6370.4 ± 483.3	6106.7 ± 777.1	6240.7 ± 555.5	5119.8 ± 578.5	**< 0.001**
**Cognitive scores**					
MMSE	28.4 ± 0.69	28.5 ± 0.85	26.11 ± 1.83	22.2 ± 2.48	**< 0.001**
Forward digit span	5.1 ± 1.12	5.1 ± 0.99	5.2 ± 1.13	4.4 ± 0.84	0.289
Backward digit span	5.8 ± 4.19	4.2 ± 1.13	4.2 ± 1.47	3 ± 0.94	0.07
FCSRT, *sum of FRs*	34.5 ± 5.2	30.6 ± 7.15	15.4 ± 3.43	4 ± 2.86	**< 0.001**
FCSRT, *sum of TRs*	47.7 ± 0.7	46.2 ± 1.68	36.3 ± 6.3	13 ± 8.83	**< 0.001**
FCSRT, *free DR*	13.7 ± 1.28	11.4 ± 2.27	4.2 ± 2.3	0.6 ± 1.07	**< 0.001**
FCSRT, *total DR*	16 ± 0	15.8 ± 0.63	11.6 ± 3.77	4.2 ± 3.19	**< 0.001**
FCSRT, *ISC*	98.4 ± 4.41	90.9 ± 8.48	61.9 ± 17.56	20.8 ± 18.02	**< 0.001**
Doors test (part a)	10.3 ± 1.49	11.2 ± 1.03	7.5 ± 2.41	5.7 ± 1.56	**< 0.001**
Doors test (part b)	5.8 ± 2.09	5.6 ± 1.95	4.4 ± 2.17	2.4 ± 1.77	**0.002**

Values are presented as mean ± SD (standard deviation). Statistically significant group effects are indicated in bold.

Group effects were assessed with ANOVA test for continuous variables and with $\chi^{2}$ (Pearson) for categorized indices. Normal values of *CSF Aβ:* > 381 pg/mL. Normal values of *CSF tau:* < 437 pg/mL. Normal values of *CSF p-tau*: < 61 pg/mL.

*HE* healthy elders, *SCD* subjective cognitive decline, *aMCI* amnestic mild cognitive impairment, *AD* Alzheimer’s disease with dementia, *GDS* geriatric depression scale (short form), *MMSE* mini mental state examination, *CDR* clinical dementia rating scale, *CSF* cerebrospinal fluid, *p-tau* phosphorylated tau, *sMRI* structural magnetic resonance imaging, *WHV* whole hippocampal volume, *FCSRT* free and cued selective reminding test, *FRs* free recalls (/48), *TRs* total recalls (/48), *DR* delayed recall (/16), *ISC* index of sensitivity of cueing, *na* not available.

All participants underwent a comprehensive neuropsychological evaluation including, among others, the evaluation of episodic/working memory and global cognition through the mini-mental state examination (MMSE, Table 1). Ongoing electrophysiological brain activity was then recorded in two consecutive 5-min resting-state sessions^39^ (eyes opened, fixation cross) using whole-scalp MEG. Hybrid PET-MR imaging for cerebral FDG-PET and high-resolution 3D T1-weighted structural cerebral MRI (3 T) were administered just after MEG.

### Network states of MEG power activation or deactivation

We inferred RSN state dynamics from MNE-reconstructed MEG signals (across all subjects and sessions) using the HMM approach^33^. Whole-brain wideband (4–30 Hz) source power envelopes were reduced to 8 transient recurrent states. This HMM along with the Viterbi algorithm returned a binary time series of state activation/deactivation associated with each state^40^. The partial correlation of these time series with brain envelope signals allowed disclosing the topographical distribution of state power associated with each state^33^. This correlation measures the degree of regional power increase/activation or decrease/deactivation during state visits. High positive (respectively negative) correlation indicated increased (respectively decreased) power envelope when the brain visited that state.

**Figure 1:**
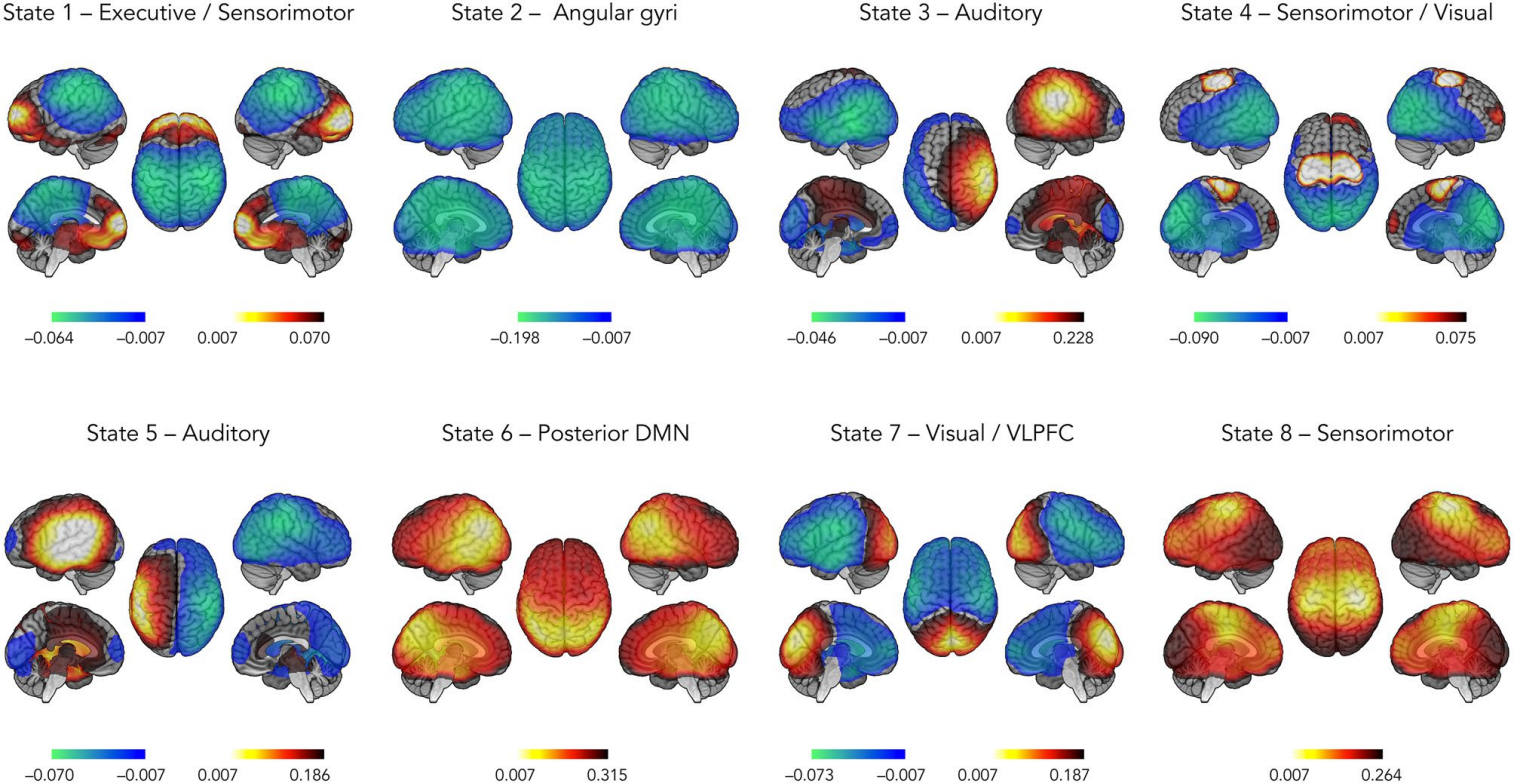
State power topography of the 8 HMM transient states. The red scale indicates positive correlation values between the envelope and the state time course (i.e., a power increase while visiting that state), whereas the blue scale indicates negative correlation values (i.e., a power decrease). *DMN* default-mode network, *VLPFC* ventrolateral prefrontal cortex.

Figure 1 presents the state power maps (see “Materials and methods” section for a description of the statistical threshold applied) of the 8 HMM transient states. Topographically speaking, State 1 involved a network configuration in which there was, upon visit, a power increase in the so-called executive network and a concomitant power decrease in the sensorimotor network. State 2 was characterized by a power decrease in bilateral angular gyri. State 3 featured increased power in the right auditory cortex and decreased power in the left lateral temporal cortex, whereas State 5 was characterized by the opposite pattern (i.e., increased power in left auditory cortex and decreased power in the right posterior temporal cortex). States 4 and 8 were marked by a power increase in the sensorimotor network, coupled with a power decrease in visual cortices for State 4. State 6 was characterized by an increase in power in brain regions corresponding to the posterior DMN (i.e., bilateral TPJs and precunei). Finally, there was increased power in visual cortices and decreased power in the ventrolateral prefrontal cortex (VLPFC) in State 7.

### Detection of altered state temporal properties

The state activation/deactivation signals also allow computing relevant state temporal parameters such as mean lifetime (MLT, i.e., the mean time spent in each state on a single visit), fractional occupancy (FO, i.e., the fraction of the total recording time that the brain spent in each state) and mean interval length (MIL, i.e., the mean time interval between two visits in the same state)^33^. Group-level (i.e., healthy elders, SCD, aMCI and AD) differences for temporal parameters were assessed using non-parametric ANOVA (Kruskal–Wallis) with Tukey’s post-hoc tests on ranks. Significance was set at *p* < 0.05 Bonferroni corrected for the number of independent states.

**Figure 2:**
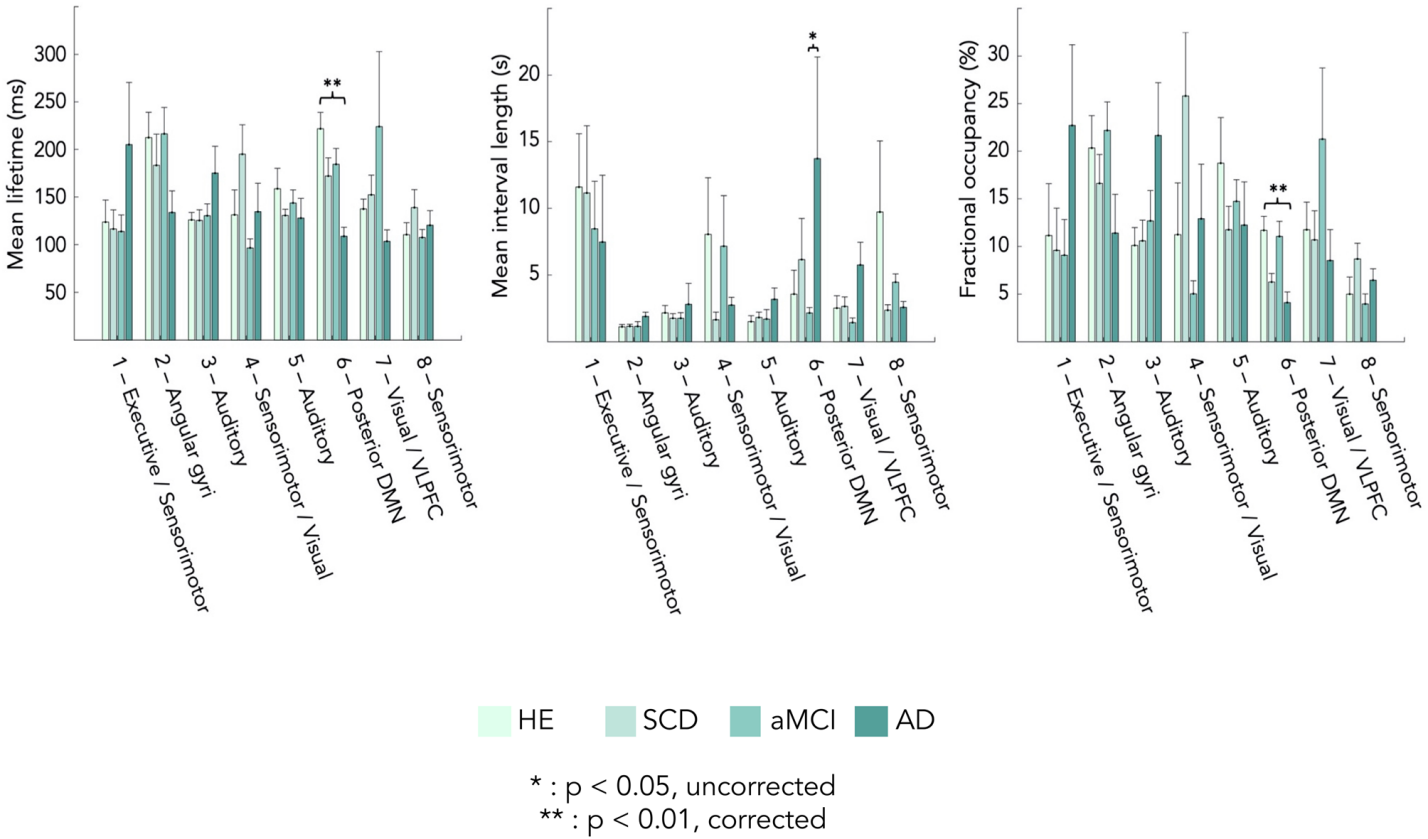
Group differences in state temporal parameters. From left to right: mean lifetime (MLT), mean interval length (MIL) and fractional occupancy (FO). *HE* healthy elders, *SCD* subjective cognitive decline, *aMCI* amnestic-mild cognitive impairment, *AD* Alzheimer’s disease with dementia, *DMN* default-mode network, *VLPFC* ventrolateral prefrontal cortex. *p < 0.05, uncorrected; **p < 0.01, Bonferroni corrected for 7 independent states. Standard error is represented above each bar.

Figure 2 displays the temporal parameters assessing the transience and stability of each state. Overall, MLT across the 8 states varied between 100 and 225 ms, which is in line with previous MEG envelope HMM studies^33, 34, 36, 37^. The only significant group effects emerged for MLT and FO of the posterior DMN activation state (State 6). The effect on MLT was explained by patients with AD spending significantly less time in that state than healthy elders (*p* < 0.001, Bonferroni corrected; healthy elders, 216.88  ± 54.56 ms; AD patients, 109.24  ± 34.85 ms). Differences with SCD (176.13  ± 61.96 ms) and MCI (185.85  ± 55.46 ms) patients did not reach significance, even at *p* < 0.05 uncorrected. Likewise, FO in the posterior DMN state was significantly lower in patients with AD than in healthy elders (*p* < 0.01, Bonferroni corrected; healthy elders, 11.78 ± 4.95%; AD patients, 4.55 ± 4.07%), but not significantly lower for SCD (6.55% ± 3.00%) and MCI (11.28 ± 5.06%) patients. No significant group differences emerged for MIL at the corrected level. A tendency for higher MIL in between visits to State 6 in the AD group was observed, but only reached uncorrected significance against the MCI group probably due to large inter-subject variability among AD patients (Fig. 2; healthy elders: 3.57  ± 5.64 s, SCD: 6.15  ± 9.75 s, MCI: 2.15  ± 1.29 s; AD: 13.73  ± 24.13 s). Overall, these findings suggest that AD is associated with a significant decrease in the stability of posterior DMN dynamics compared to healthy elders.

Based on these results, we selected MLT and FO of the posterior DMN activation State 6 for further correlation analyses with (1) cognitive test scores (Table 1), (2) the whole hippocampal volume (Table 1), and (3) regional brain glucose metabolism.

### Correlation between State 6 temporal parameters and cognitive test scores

Correlation analysis between State 6 temporal parameters (MLT and FO) and cognitive test scores (episodic and visual memory, working memory and MMSE) was performed using multiple regression across the 40 participants but with group of participants (i.e., healthy elders, SCD, aMCI, AD) set as covariates of no interest. This analysis disclosed no significant relationship (all *p*s > 0.05, corrected). Thus, no conclusive link could be drawn between the temporal instability of posterior DMN activation and cognitive functioning.

### Hippocampal volume and correlation with State 6 temporal parameters

The left and right hippocampal grey matter volumes were identified by tissue segmentation of participants’ structural MRI using FreeSurfer v6.0 (Martinos Center for Biomedical Imaging, Massachusetts, USA). Patients with AD has smaller whole hippocampal volumes (*p* < 0.01, Bonferroni corrected; AD patients, 5119.87 ± 578.57 mm^3^) than healthy elders (6370.44 ± 483.3 mm^3^) and than SCD (6106.78 ± 777.15 mm^3^) or aMCI (6240.79 ± 555.51 mm^3^) patients. No significant change was observed between healthy elders and patients with SCD nor aMCI (*p* > 0.05, corrected).

Correlations between MLT and FO of State 6 and whole hippocampal volume were not significant when computed across the 40 participants with group of participants as covariates of no interest (MLT: r = − 0.13, *p* = 0.43; FO: r = 0.25; *p* = 0.13).

### Regional cerebral metabolism and correlation with State 6 temporal parameters

**Figure 3:**
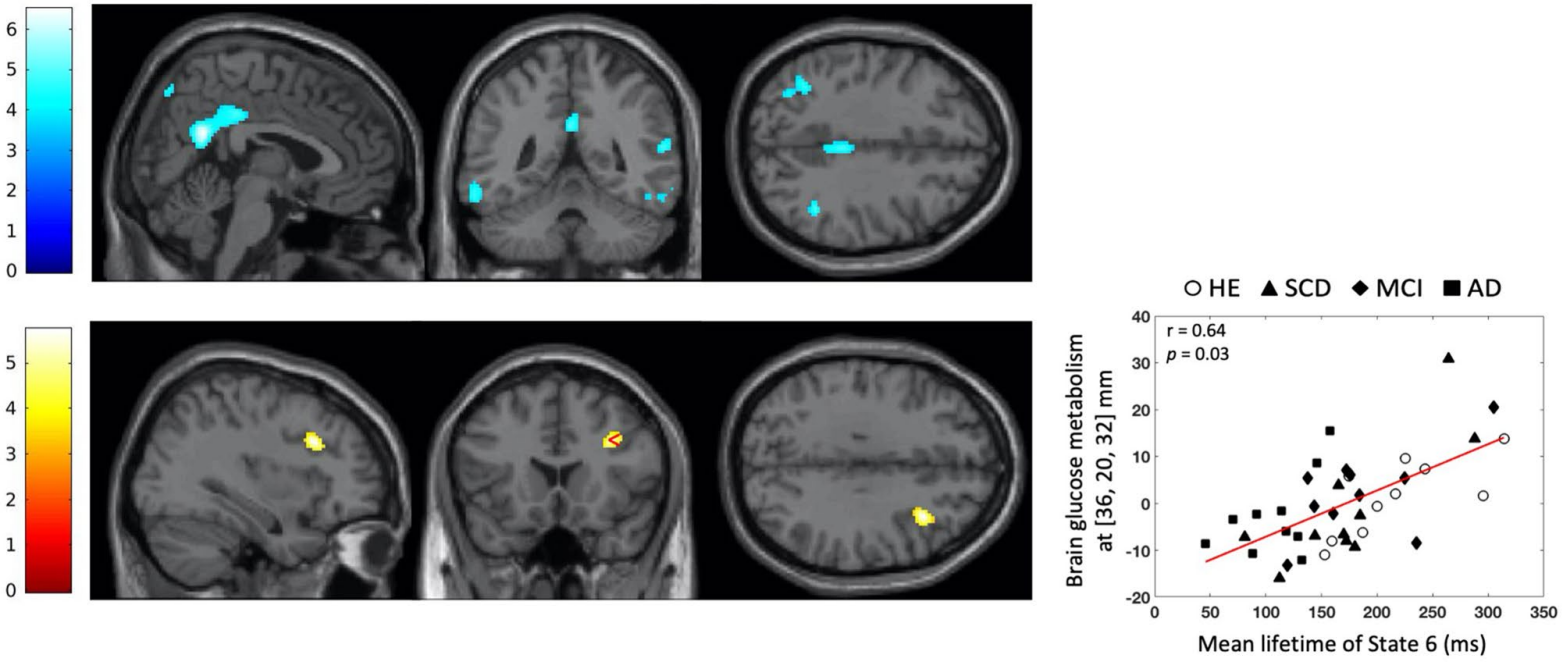
Statistical parametric *T* maps showing a significant reduction in regional glucose consumption in AD patients compared with healthy elders (Top) and a significant positive correlation between mean lifetime of State 6 and regional glucose consumption across the 40 participants with group as covariates of no interest (Bottom). Images are thresholded at *p* < 0.001 uncorrected with a cluster threshold at 100 voxels for visualization purpose. The color scales represent the T-statistic of the significant voxels.

Voxel-based subtractive analyses of FDG-PET data first investigated using in SPM12 (Wellcome Centre for Neuroimaging, London, UK) the brain regions showing increase or decrease in glucose consumption between healthy elders and patients with SCD, aMCI or AD. These analyses demonstrated that patients with AD had a significant reduction in glucose consumption in the precunei, bilateral angular gyri, and right inferior temporal region compared with healthy elders (*p* < 0.05 with family-wise error (FWE) correction at the cluster level; see Fig. 3, top). No significant difference was observed between healthy elders and patients with SCD or aMCI.

We then performed statistical parametric correlation maps of State 6 temporal parameters and regional brain glucose metabolism. We identified a positive correlation (computed once again across the 40 participants but including group-specific covariates of no interest) between MLT and the metabolic activity of the right dorsolateral prefrontal cortex (DLPFC) (*p* = 0.034, FWE-corrected, peak correlation at MNI coordinates [36, 20, 32] mm; see Fig. 3, bottom). Of note, this result would not survive a Bonferroni correction for the two correlation tests performed (i.e., with MLT and FO of State 6). However, MLT and FO are likely somewhat correlated, so the Bonferroni method would lead to overly conservative statistics.

## Discussion

This study evidenced less frequent visits and less time spent in a transient recurrent state of activated posterior DMN in patients with AD than in matched healthy elders. No difference in transient resting-state brain dynamics was found between patients in pre-clinical (i.e., SCD) or pre-dementia (i.e., MCI) stages and either the patients with AD or the healthy elders. The time spent by the participants in the activated posterior DMN state was positively correlated with glucose consumption in right DLPFC, but did not correlate with the whole hippocampal volume, memory and global cognition scores.

The 8 transient recurrent HMM states disclosed in our participants exhibited spatial and temporal patterns rather similar to those previously described^33, 34, 36, 37, 41^. Importantly, we also found one state (State 6) characterized by activated bilateral TPJs and precunei when visited, corresponding to an activated posterior DMN state. Involvement of posterior midline cortices such as the precuneus was not disclosed in previous MEG HMM studies that identified similar DMN states^33, 34^. In particular, it was not observed in the DMN state reported in the MEG envelope HMM study of AD^34^. This is presumably related to the use of MNE rather than beamforming for source reconstruction in this study, as it was shown that the former is better suited to image midline posterior cortices in functional integration studies^38^.

Patients with AD showed a dynamical destabilization of this posterior DMN activation characterizing State 6. There was a significant reduction in MLT and FO of that state in patients with AD compared with matched healthy elders (along with a trend for higher MIL in AD patients). These findings are in line with those of a previous MEG HMM study performed in AD patients^34^, except that we further identified altered dynamics in the bilateral precunei. Furthermore, we investigated here a population of AD patients with established abnormal amyloid and tau protein levels in the CSF, reduced whole hippocampal volume and reduced cerebral glucose metabolism in brain areas that are pathognomonic of AD. Our results therefore strongly reinforce the hypothesis that, compared to healthy elders, AD is characterized by a significant reduction in the temporal stability of power activity within the posterior DMN. Considering the pathognomonic decrease in (static) resting-state functional connectivity within the DMN typically found in AD patients (for a review, see, e.g., Ref.^42^), this finding suggests that established AD-related dementia is accompanied by a reduction in both DMN functional integration and the stability of its dynamic activation.

The time spent by participants in the activated posterior DMN state did not correlate with memory/global cognition scores (episodic and visual memory, working memory and MMSE scores), nor with the whole hippocampal volume. This finding suggests that the aberrant AD-related destabilization of resting-state posterior DMN activation may not be directly linked to cognitive impairment, nor to the decrease in hippocampal volume typically observed in AD patients. This lack of conclusive correlation with the cognitive profile of AD patients was also observed in a rsfMRI study of dynamic functional integration^43^. A specific hypothesis for this is further developed below.

By contrast, a positive correlation was observed between the time spent by participants in the activated posterior DMN state and glucose consumption in the right DLPFC, demonstrating that the higher the dynamical stability for that state, the higher is the glucose metabolism in this brain area. The metabolic activity of the right DLPFC is therefore linked to the dynamical stability of the posterior DMN across groups of participants, and this relationship is preserved in patients with AD. This finding might be related to previous empirical demonstration that transcranial direct current stimulation of the DLPFC (1) modulates functional connectivity within DMN components^44, 45^, and (2) increases the propensity to mind wander^46, 47^ (although debated, see Ref.^48^), a brain state during which DMN activity is particularly high^49, 50^. Activity within nodes of the executive control network might therefore modulate the dynamic stability of the posterior DMN, but future studies in larger groups of participants are mandatory to confirm that statement.

A key issue associated with our findings is to determine whether the AD-related changes in transient DMN dynamics are linked to the possible effects of pathological aging on intrinsic (i.e., task-independent) functional brain integration or on extrinsic (i.e., task-dependent) spontaneous cognitive processes, or on their complex interactions. Indeed, whether spontaneous brain activity and functional integration reflect intrinsic neural processes (e.g., maintenance of homeostasis or the integrity of anatomical connections) or extrinsic neural processes (e.g., mind wandering), or both, remains an open question (for a review, see, e.g., Ref.^51^). As certain subtypes of spontaneous cognitive processes are detectable in time-varying functional connectivity measurements^51^, it could be hypothesized that AD-related changes in transient resting-state DMN activation dynamics observed in this study might pertain to the emerging literature about changes in the occurrence of mind wandering episodes and in the content/type of spontaneous cognitive processes observed in pathological aging^52–54^. Elders indeed have a tendency to experience less mind wandering episodes and with different cognitive contents than young adults^55–58^, with a further decline in AD patients^52^. AD-related alterations in spontaneous cognitive processes seem indeed highly plausible considering the key role of the DMN in mind wandering in particular and spontaneous cognition more generally, and the peculiar DMN vulnerability (amyloid-β and tau protein deposition, atrophy, hypometabolism) in AD (for a review, see Ref.^59^). Based on those considerations, the destabilization of posterior DMN activations at rest observed in our sample of patients with AD might represent an electrophysiological correlate of the decrease in mind wandering episodes previously reported in AD. The absence of link between rather stable (i.e., across hours, days) cognitive deficits^60, 61^ and transient (a few hundred ms) resting-state posterior DMN dynamics might bring additional support to this hypothesis. Further studies combining behavioral and electrophysiological -and eventually metabolic-brain data along the AD continuum are therefore mandatory to clarify this issue.

To our knowledge, this study is the first to investigate the dynamic properties of resting brain activity using an HMM approach applied to MEG power envelope signals from potentially preclinical and predementia stages of AD (i.e., in SCD and aMCI patients). However, analyses failed to identify any significant difference in HMM state temporal parameters between these groups and healthy elders or AD patients. A possible explanation is that the dynamic electrophysiological abnormalities in the DMN are too subtle to be detectable in preclinical and prodromal stages of AD, and would rather progress sharply at the latest stage. As a consequence, our relatively small sample size could induce a lack of significant sensitivity.

We also cannot exclude the hypothesis that our samples of patients with SCD and aMCI were mostly composed of patients who will not evolve towards AD, due to the absence of amyloid-/tau-PET or CSF investigations in these participants and the negative structural/metabolic group-level neuroimaging findings (although again the small sample size may affect sensitivity). Indeed, the evolution of preclinical and predementia stages is extremely variable between individuals (see, e.g., Ref.^62^). MCI and SCD stages are heterogeneous conditions that may be caused by different pathologies (see, e.g., Refs.^63–66^), so it can be expected that the majority of SCD and MCI patients included in our study will actually never progress to AD. Still, we have applied strict inclusion criteria to prevent the cognitive profile from being better explained by other types of dementia or mental disorders than AD (e.g., vascular dementia, depression, etc.). To confirm the robustness of our findings, further HMM-MEG studies focusing on SCD and MCI patients should therefore include larger patients’ cohorts, with a longitudinal design to assess the progression, stabilization or normalization of cognitive impairments in SCD or MCI patients, and systematic measurements of core AD biomarkers.

The present study suffers from several methodological limitations. First, as mentioned above, the sample size of each group (healthy elders, SCD, aMCI and AD) was rather modest. This was sufficient to demonstrate similar HMM results than the previous HMM MEG study performed in AD^34^, using the same number of AD patients but with established abnormal amyloid and tau protein CSF levels and neuroimaging markers of AD. This replication therefore suggests that the observed differences in transient resting-state DMN dynamics between AD patients and healthy elders are robust. Still, this modest sample size may have impacted our sensitivity to detect possible subtle differences between pre-clinical/pre-dementia stages and healthy elders or AD patients. Further studies including large numbers of participants along the AD continuum are therefore mandatory to confirm our findings. Second, as mentioned before, SCD and MCI stages are heterogeneous conditions characterized by different possible progressions to AD or non-AD dementia, stabilization or even normalization of their cognitive profile^62^. That could partially explain the lack of significant results for these preclinical and predementia stages. Indeed, our study is cross-sectional and, consequently, the accurate percentage of SCD and MCI patients who will develop AD in few years is unknown. Future longitudinal investigations are needed to explore the validity of these results. Third, a refinement of the criteria defining these preclinical and predementia stages, notably with the measurement of CSF biomarkers (i.e., amyloid-beta, total-tau and phosphorylated-tau), could be particularly useful in that context. Indeed, a previous study showed that CSF biomarker score provided a good estimate of the risk of MCI to AD dementia conversion up to 6 years in comparison with lower CSF biomarker score^67^. Finally, recent works have shown that the use of cryogenic MEG systems induced an underestimation of the level of frontal functional integration due to inhomogeneities in the MEG sensor-brain distance^51, 68^. We cannot therefore totally exclude that some AD-related changes have been underestimated in those brain regions due to a lower signal to noise ratio. The use of on-scalp MEG based on optically pumped magnetometers, which have been demonstrated to be usable for neuromagnetic investigations in humans, should therefore be privileged in the future^69^.

In summary, this study demonstrated that abnormalities in fast brain network activation dynamics can be identified in AD using HMM analysis of resting-state MEG power envelopes. Results suggested that AD-related alterations of posterior DMN activity may be considered as an additional electrophysiological correlate of the AD-related synaptic and neural dysfunction. Whether these alterations reflect AD-related changes in mind wandering episodes remains an open key question.

## Materials and methods

### Participants

Ten healthy elders, 10 patients with SCD *plus* (see “Diagnostic criteria” below), 10 with aMCI and 10 with typical AD were included in this study. All participants gave their written informed consent prior to their participation in this study. The Ethics Committee of CUB Hôpital Erasme (P2017/427, Brussels, Belgium) approved this study. All experiments were performed in accordance with relevant guidelines and regulations.

General exclusion criteria were previous other neurological or psychiatric disorders, a modified Hachinski Ischemic Score ≥ 4 (HIS)^70^, chronic use of psychotropic drugs or alcohol, insufficient level in French language, age younger than 55 or older than 90 years, and severe visual or hearing impairment. All individuals had at least 6 years of education and the majority of them were right-handed (except for one healthy subject who was left-handed, and one healthy subject and one aMCI who were ambidextrous) according to the Edinburgh handedness inventory^71^.

### Clinical evaluation

All participants were initially screened for exclusion criteria. Then, the clinical evaluation included the minimal mental scale examination (MMSE)^72^, the short form of the geriatric depression scale (GDS)^73^, the neuropsychiatric inventory (NPI)^74^, the Pittsburgh sleep quality index (PSQI)^75^ and a questionnaire assessing the quality of participants' previous night of sleep (adapted from^76^). An exhaustive anamnesis and a hetero anamnesis were conducted with all patients and their caregivers in order to learn more about their medical and psychiatric history. Furthermore, the clinical dementia rating (CDR)^77^, and the basic and instrumental activities of daily living (BADLs and IADLs)^78^ questionnaires were used to evaluate the severity of symptoms and their potential impact on everyday life. Each participant also underwent a comprehensive neuropsychological assessment comprising the evaluation of (1) verbal episodic memory with the free and cued selective reminding test (FCSRT)^79, 80^, (2) visual episodic memory using the Doors and People test (only the Doors part was administered)^81^, (3) working memory by the forward and backward digit span test (Wechsler Memory Scale, WMS-III), (4) language abilities with the ExaDé naming test^82^, (5) executive functions and, more specifically, the flexibility sub-component using the phonemic/letter and categorical fluency test^83^, as well as the trail making test (part B)^84^ and the inhibition sub-component with the interference part of the Stroop color and word test^85^, (6) visuo-constructive abilities with the Rey–Osterrieth figure copy^86^ and finally, (7) speed processing and visual attention with the trail making test (part A)^84^.

### Diagnostic criteria

Healthy elders had a normal neuropsychological performance and no subjective memory complaint. SCD patients were defined following Jessen’s criteria including : (1) self-experienced persistent decline in cognitive capacity in comparison with a previously normal status and unrelated to an acute event, (2) normal age, gender and education-adjusted performance on standardized neuropsychological tests, (3) not to be MCI or AD as per the criteria described below, and (4) subjective cognitive decline cannot be explained by a psychiatric or neurologic disease, medical disorder or substance use^9^. SCD *plus* were defined with subjective decline in memory rather than other domains of cognition, the onset of SCD within the last 5 years, the age at onset of SCD ≥ 60 years, concerns and feelings of worse performance than others of the same age group (evaluated here with the GDS-short form question: *“Do you feel you have more problems with your memory than most ?”*^73^). The aMCI subjects were classified according to the core clinical criteria developed by the National Institute on Aging-Alzheimer’s Association (NIA-AA) workgroups^10^: (1) concerns regarding a change in cognition, (2) an impairment in one or more cognitive domains, (3) preservation of independence in functional abilities, and (4) to be not demented. Furthermore, according to their main memory impairment, they were all classified with the amnestic profile of MCI^87^. Finally, the new criteria of NIA-AA^88^ were used to define AD patients with dementia following the amyloid deposition, pathologic tau, and neurodegeneration [AT(N)] classification that groups different biomarkers by the pathologic process. Indeed, all AD patients had abnormal Aβ42 (A +), phosphorylated tau (T +) and total-tau (N +) following the lumbar puncture analysis with measurement of the CSF core biomarkers^88^. Typical profile of AD patients was characterized by greater deficit of verbal episodic memory than other cognitive functions. Indeed, one study showed that 79.6% patients with typical AD cognitive profile have an autopsy-confirmed diagnosis compared to only 20.4% for atypical AD group^89^.

### Multimodal neuroimaging data acquisition

In all participants, neuroimaging data acquisition was performed on the same day. MEG data were acquired first, followed by PET-MR data.

Neuromagnetic data were acquired (two sessions of 5 min, sitting position, eyes open with fixation cross, online band-pass filter: 0.1–330 Hz, sampling rate: 1000 Hz) by using a 306 channel whole-scalp MEG system (Triux, MEGIN, Helsinki, Finland) placed inside a light-weight magnetically shielded room (Maxshield, MEGIN, Helsinki, Finland; see Ref.^90^ for more details) at the CUB Hôpital Erasme (Brussels, Belgium). The position of participants’ head was recorded continuously inside the MEG helmet by using four head tracking coils. Coils’ location and approximatively 300 head points were determined following the anatomical fiducials with an electromagnetic tracker (Fastrak, Polhemus, Colchester, Vermont, USA). Furthermore, eye movements or blinks and cardiac artifacts were captured with bipolar electrodes.

T1-weighted MRI (Repetition Time/Echo Time/Flip Angle: 8.3 ms/3.1 ms/12°, Time of Inversion: 450 ms, Field of View: 24 cm × 24 cm, resolution: 1 mm × 1 mm × 1 mm) and FDG-PET data acquisitions were performed simultaneously on a hybrid 3 T SIGNA PET-MR scanner (GE Healthcare, Milwaukee, Wisconsin, USA) at the Service of Nuclear Medicine (CUB Hôpital Erasme, Brussels, Belgium). All participants fasted for at least 4 h, were awake in an eye-closed rest and received an intravenous bolus injection of 2–5 mCi (74–185 MBq) of FDG before PET-MR data acquisition. The time interval between FDG injection and the start of data acquisition was 40 min and the total scan duration was 20 min. PET data were reconstructed using the fully 3D iterative reconstruction algorithm VUE Point FX-S, which takes into account the time-of-flight information and the correction for the point spread function of the system. The algorithm was configured with 10 iterations, 28 subsets and a standard Z-axis filter cut-off at 4 mm. The photons’ attenuation specific of the PET acquisition was corrected with a MRI-based map (MRAC) acquired simultaneously. PET images were displayed in a 256 × 256 × 89 matrix format, with a slice thickness of 2.78 mm. The reconstructed files were downloaded in their original format (DICOM, ECAT, Interfile) for meta-information and converted in NIfTI format to be finally used for analysis.

### Neuroimaging data preprocessing

MEG data were filtered offline using temporal signal space separation to remove external interferences and nearby sources of magnetic artifact, but also compensate for head movements^91^. Cardiac, ocular and system artifacts were visually identified and eliminated using an independent component analysis (FastICA algorithm with dimension reduction to 30 components, hyperbolic tangent nonlinearity function)^92^ of the filtered data (off-line band-pass filter: 0.1–45 Hz).

The MEG forward model was computed on the basis of participants' 3D T1-weighted cerebral MRI, which was anatomically segmented beforehand using the FreeSurfer software (version 6.0; Martinos Center for Biomedical Imaging, Massachusetts, USA). Of note, this segmentation procedure also provided the right and left whole hippocampal grey matter volumes used for further correlation analyses. FreeSurfer is classically used to obtain hippocampal grey matter volumes in the field of AD^93–95^. The co-registration between MEG and MRI data was then performed using the 3 fiducial points (nasion and auricular points) for first estimation and the head-surface points to manually refine the surface co-registration (Mrilab, Elekta Oy, Helsinki, Finland). Then, a volumetric and regular 5-mm source grid was built using the Montreal Neurological Institute (MNI) template and non-linearly deformed onto each participants’ MRI with the Statistical Parametric Mapping Software (SPM12, Wellcome Centre for Neuroimaging, London, UK). The three-dimensional MEG forward model associated with this source space was computed using a one-layer Boundary Element Method as implemented in the MNE-C suite.

The preprocessing of FDG-PET images was performed with SPM8 (www.fil.ion.ucl.ac.uk/spm) and spatially normalized using a specific FDG-PET aging and dementia template^96^. Data were then smoothed with a FWHM 8-mm Gaussian kernel.

### MEG source reconstruction

A band-specific MNE^97^ was applied to perform inverse modeling that was restricted to the 204 planar gradiometers. Five minutes of artifact-free noise measurement, obtained from an empty-room MEG recording filtered spatially with signal space separation^91^ and spectrally in the relevant frequency range, was recorded for the estimation of the noise covariance matrix. The regularization parameter was calculated with the consistency condition (as derived in^98^). The depth bias was corrected using a noise normalization scheme, i.e., dynamic Statistical Parametric Mapping^97^. Each estimated three-dimensional source was projected onto its direction of maximum variance, filtered in the 4–30 Hz band, and Hilbert transformed to extract their wide-band envelope signal fed to the HMM analysis.

### Hidden Markov modeling

We thoroughly followed the pipeline described in Refs.^33, 99^ implemented in GLEAN (https://github.com/OHBA-analysis/GLEAN), except for the use of MNE as inverse model rather than a Beamformer. The number of transient states (*K*) was set to 8 for consistency with previous MEG envelope HMM studies^33, 99^. The 8-state HMM was inferred from the wide-band (4–30 Hz) source envelope signals. These envelopes were first downsampled at 10 Hz using a moving-window average with 75% overlap (100 ms wide windows, sliding every 25 ms), leading to an effective downsampling at 40 Hz. Envelope data were demeaned and normalized by the global variance and then concatenated temporally across participants to design a group-level HMM analysis. Group-concatenated envelopes were finally pre-whitened and reduced to 40 principal components. The HMM per se was then applied to determine dynamic states of envelope covariance, along with the Viterbi algorithm to obtain binary time series of state activation/deactivation under the constraint of temporal exclusivity^40^. These binary signals allowed to determine individual estimates of several state temporal parameters such as the MLT (i.e., mean duration of time intervals of active state), the FO (i.e., total fraction of time during which the state is active) and the MIL (i.e., mean duration of time intervals of inactive state).

Rank-based ANOVA (Kruskal–Wallis) was used to detect group effects (healthy elders, SCD, aMCI, AD) on state-specific temporal properties. We favored this non-parametric test because of its reliance against outliers, which sometimes arise among HMM state temporal parameters when, e.g., one or a few subjects scarcely visit one state. Significance was set to *p* < 0.05 Bonferroni corrected for the number *K*-1 = 7 of independent states (one state being dependent upon the others due to the temporal exclusivity constraint). Tukey’s post-hoc testing on data ranks allowed to identify group differences explaining significant ANOVA.

State power maps were built as the partial correlation between HMM state activation/deactivation time series and group-concatenated source envelope signals. These correlations assessed state-specific power changes upon state activation. These maps were thresholded statistically by applying a two-tailed parametric correlation test at *p* < 0.05 against the null hypothesis that Fisher-transformed correlations following a Gaussian with mean zero and standard deviation $1/\sqrt {N_{tdof} - K - 3}$. The number *N*_*tdof*_ of temporal degrees of freedom was calculated as one-quarter of the total number of time samples to take into account the overlap of 75% in the envelope downsampling. The critical *p* value was Bonferroni corrected with number of independent HMM states (i.e., 7) multiplied by the number of spatial degrees of freedom in MNE maps (estimated from the rank of the MEG forward model^98^).

### Differences in cognition, whole hippocampal volume and regional metabolism between groups of participants

The differences in cognitive scores (Table 1) among the four groups (i.e., healthy elders, SCD, aMCI and AD) were first evaluated by analysis of variance (ANOVA) using Bonferroni post hoc analysis. A *p* value < 0.05 was considered significant.

Then, we searched for significant differences in whole hippocampal volume. For that purpose, statistically significant group differences based on ANOVA (*p* < 0.05) were explored using Bonferroni post hoc analysis.

Finally, we performed subtractive voxel-based analysis of FDG-PET data as done in previous studies from our group^100–103^. We used SPM12 (https://www.fil.ion.ucl.ac.uk/spm/, Wellcome Trust Centre for Neuroimaging, London, UK) to construct general linear models (GLM) of the preprocessed FDG-PET data of healthy elders and patients with SCD, aMCI or AD taken as separate groups (i.e., one GLM per group of patients). Proportional scaling was applied beforehand to remove inter-subject variation in global brain metabolism and an explicit FDG mask was used to restrict the analyses inside the brain. Separate mass-univariate *T* contrasts then searched, throughout the brain, for regions showing significant decrease or increase in metabolism between healthy subjects and each group of patients. Results were considered significant at *p* < 0.05 FWE-corrected unless otherwise stated.

### Correlation between temporal properties of HMM states and cognition, whole hippocampal volume, and regional brain metabolism

Correlation analyses with altered state temporal parameters (i.e., disclosing a significant group effect) aimed at obtaining novel insights into the pathophysiology of their alterations in the context of AD.

First, the Pearson correlation coefficient was estimated (across the 40 participants, using multiple regression with groups of participants (i.e., healthy elders, SCD, aMCI, AD) as covariates of no interest) between altered state temporal parameters and cognitive scores. More specifically, the scores included in this analysis were : verbal episodic memory as assessed using Delayed Recall after 20 min, sum of free recalls, sum of total recalls and the index of sensitivity of cueing ([“sum of the 3 total recalls” – “sum of the 3 free recalls”]/[48 – “sum of the 3 free recalls”], defined by Ref.^104^) from Ref.^80^, the visual episodic memory with the doors and people test (only the doors part was administered)^81^, working memory with forward and backward digit span tests (Wechsler Memory Scale, WMS-III) and finally, global cognition defined by MMSE score^72^. The results were considered significant at *p* < 0.05 Bonferroni corrected for multiple comparisons following the number of cognitive scores considered.

A similar Pearson correlation analysis was performed with the whole hippocampal volume, computed as the sum of left and right whole hippocampal grey matter volumes obtained from structural MRI segmentation using FreeSurfer (see above). Our focus on the hippocampus was driven by that hippocampal atrophy is a well-recognized feature of AD and an established neuroimaging marker^10, 105^.

Finally, a mass-univariate version of Pearson correlation analysis was used to explore the relationship between state temporal parameters and regional cerebral glucose metabolism. We used SPM12 (https://www.fil.ion.ucl.ac.uk/spm/, Wellcome Trust Centre for Neuroimaging, London, UK) to construct two (i.e., one for MLT and one for FO) GLMs of the preprocessed FDG-PET data of all participants (40 scans), with the mean-centered state temporal parameter (i.e., MLT or FO) as covariate of interest and group of participant (i.e., healthy elders, SCD, aMCI, AD) as covariates of no interest. Proportional scaling was applied beforehand to remove inter-subject variation in global brain metabolism and an explicit FDG mask was used to restrict the analyses inside the brain. Separate *T* contrast analyses then searched, throughout the brain, for regions showing significant positive or negative correlations with state temporal parameters. As such, this analysis is an adaptation of the previously developed psycho-, physio-, or patho-physiological interaction analyses^100, 106^ to a specific experimental factor, i.e., state temporal parameters. Results were considered significant at *p* < 0.05 FWE-corrected unless otherwise stated.

## Data Availability

The datasets analyzed during this study are available from the corresponding author upon reasonable request and after approval by institutional authorities.
